# Isolation and characterization of canine perivascular stem/stromal cells for bone tissue engineering

**DOI:** 10.1371/journal.pone.0177308

**Published:** 2017-05-10

**Authors:** Aaron W. James, Xinli Zhang, Mihaela Crisan, Winters R. Hardy, Pei Liang, Carolyn A. Meyers, Sonja Lobo, Venu Lagishetty, Martin K. Childers, Greg Asatrian, Catherine Ding, Yu-Hsin Yen, Erin Zou, Kang Ting, Bruno Peault, Chia Soo

**Affiliations:** 1 Department of Pathology, Johns Hopkins University, Baltimore, Maryland, United States of America; 2 UCLA and Orthopaedic Hospital Department of Orthopaedic Surgery and the Orthopaedic Hospital Research Center, Los Angeles, California, United States of America; 3 Division of Growth and Development and Section of Orthodontics, School of Dentistry, University of California Los Angeles, Los Angeles, California, United States of America; 4 Center for Cardiovascular Science and MRC Center for Regenerative Medicine, University of Edinburgh, Edinburgh, United Kingdom; 5 Rehabilitation Medicine Clinic, UWMC, Seattle, Washington, United States of America; 6 Division of Plastic and Reconstructive Surgery, Department of Surgery, David Geffen School of Medicine, University of California Los Angeles, Los Angeles, California, United States of America; Instituto Butantan, BRAZIL

## Abstract

For over 15 years, human subcutaneous adipose tissue has been recognized as a rich source of tissue resident mesenchymal stem/stromal cells (MSC). The isolation of perivascular progenitor cells from human adipose tissue by a cell sorting strategy was first published in 2008. Since this time, the interest in using pericytes and related perivascular stem/stromal cell (PSC) populations for tissue engineering has significantly increased. Here, we describe a set of experiments identifying, isolating and characterizing PSC from canine tissue (N = 12 canine adipose tissue samples). Results showed that the same antibodies used for human PSC identification and isolation are cross-reactive with canine tissue (CD45, CD146, CD34). Like their human correlate, canine PSC demonstrate characteristics of MSC including cell surface marker expression, colony forming unit-fibroblast (CFU-F) inclusion, and osteogenic differentiation potential. As well, canine PSC respond to osteoinductive signals in a similar fashion as do human PSC, such as the secreted differentiation factor NEL-Like Molecule-1 (NELL-1). Nevertheless, important differences exist between human and canine PSC, including differences in baseline osteogenic potential. In summary, canine PSC represent a multipotent mesenchymogenic cell source for future translational efforts in tissue engineering.

## Introduction

Mesenchymal stem/stromal cells (MSC) are a multipotent cell population with multiple applications in bone tissue engineering, including promotion of wound repair[1] and tissue regeneration[2]. Bone marrow and adipose tissue are the two major tissue sources of MSC most studied for bone tissue regeneration. However, culture-derived MSC from both tissues are accompanied by significant drawbacks for bone tissue engineering. Bone marrow mesenchymal stem/stromal cells (BMSC) come with significant impediments for clinical translation, including low stem cell frequency, harvest site morbidity, and requirement for culture derivation. In contrast, adipose tissue is abundant, accessible and readily available by routine liposuction procedures with minimal donor site morbidity[3–5]. Unfortunately, the cellular heterogeneity of the stromal vascular fraction (SVF) of adipose tissue is associated with reduced or unreliable bone forming efficacy[6, 7].

Perivascular stem/stromal cells (PSC) are a homogeneous MSC population purified by fluorescence activated cell sorting (FACS), that can be used for regenerative medicine applications without culture expansion[8, 9]. They are abundant in human white adipose tissue and are present in clinically relevant numbers (approximately 40% of the viable human SVF)[10]. PSC originate in the vessel wall[11, 12] and are composed of two distinct yet related cell populations, including pericytes (CD34-CD146+CD45-) and adventitial cells (CD34+CD146-CD45-)[12, 13]. Importantly, PSC are robustly osteogenic in culture and *in vivo*, migrate actively, stimulate angiogenesis and secrete diverse growth and differentiation factors (GDFs) to a greater degree than traditionally derived MSC[12, 14]. Compared with unpurified cells of the SVF, PSC have shown a significantly increased ability to form bone in several *in vivo* models[10, 15]. Moreover, PSC have been shown to promote *in vivo* bone regeneration across multiple small animal models, including a mouse critical-sized calvarial defect model[8], and a rat lumbar spinal fusion model[16, 17].

The commitment of MSC to an osteogenic cell fate relies on many signaling pathways and transcription factors, including: Hedgehog signaling[18–20], β-catenin dependent Wnt signaling, β-catenin independent or non-canonical Wnt signaling,[21–23] and NEL-Like Molecule-1 (NELL-1) signaling[20, 24], among others. NELL-1 is a secreted osteoinductive protein that has been studied for its ability to promote osteogenic differentiation in a relatively bone-specific manner[25–29]. NELL-1 is known to bind to the cell surface receptor Integrin β1, resulting in focal adhesion kinase (FAK) phosphorylation[30] and a cascade of intracellular signaling events that regulates the activity of Runt-related transcription factor-2 (RUNX2)[31]. NELL-1 protein has been previously observed to enhance the osteogenic differentiation of human PSC *in vitro*, as well as increase PSC-mediated bone formation *in vivo*[10]. The application of PSC with an osteoinductive protein such as NELL-1 may be an effective future combination therapy for local bone repair.

In prior studies, immunocompromised small animal models have allowed us to examine the *in vivo* bone forming potential of human PSC. In order to translate purified perivascular cell therapies into a clinical possibility, we next sought to translate these findings to a large animal model. Use of human PSC in a large animal would require use of immunomodulatory drugs that inhibit tissue repair. Thus, in the current project we sought to purify and validate the use of large animal (canine) PSC from subcutaneous adipose tissue.

The dog has several unique advantages over other model organisms. Aside from humans, canines are the most extensively studied species in medicine[32]. The recent sequencing of the canine genome[33] has revealed significant homology between human and canine genes, many of which are affected in naturally occurring diseases shared by the two species[34]. In the context of bone disorders, canines naturally suffer bone related diseases which develop similar to those in humans[35]. Thus, canines have been utilized as a translational model in a wide range of bone pathology and regeneration studies, including limb-sparing orthopaedic reconstruction[36] and distraction osteogenesis[37]. Of particular interest to the field of bone tissue engineering, models have been well-described in canine using adipose derived stem/stromal cells (ASC) for the treatment of cranial defects[38], segmental bone defects[39], radial and ulnar nonunion fractures[40], and osteoarthritis[41, 42]. Therefore, we investigated the potential of canine PSC as a prospective cell population for translational bone regeneration applications. Our study revealed predominant similarities between canine and human PSC, thus paving the way for use of canine PSC for *in vivo* efforts in bone tissue engineering.

## Materials and methods

### PSC identification, isolation and culture

Canine adipose tissue was obtained under IACUC approval from 12 mongrel, adult research animals of mixed gender (University of California, Los Angeles). Unless otherwise stated, subcutaneous tissue from the dorsum of the animal was used.

In order to test the cross reactivity of human antibodies with canine tissue, immunofluorescent detection of markers of pericytes (CD146; AbD Serotec, Raleigh, NC), adventitial cells (CD34, BD Biosciences, San Jose, CA), and endothelium (CD31, Abcam, Cambridge, MA) was performed on frozen sections of canine tissue. Canine adipose tissue was snap frozen and cryosections were obtained at -30°C. Sections were fixed in a solution of 1:1 methanol:acetone for 10 minutes at room temperature prior to immunofluorescent staining. Incubation was done at 1:100 for all antibodies at 4°C overnight. Images were captured using a Zeiss LSM 780 Confocal microscope (with Fluorescence Correlation Spectroscopy) using a 40 × or 63 × objective oil immersion lens. Next, PSC were analyzed and isolated from canine adipose tissue via flow cytometry and fluorescence activated cell sorting (FACS). Isolation and culture were performed as previously described for human PSC isolation[8, 9]. Lipoaspirate specimens were enzymatically digested to obtain the stromal vascular fraction (SVF). Next, SVF was incubated with antibodies recognizing CD146 (1:100, AbD Serotec, Raleigh, NC), CD45 (1:100), and CD34 (1:100) (BD Biosciences, San Jose, CA). The viability dye DAPI (1:1,000, Invitrogen, Carlsbad, CA) was also added prior to sorting. Cells were sorted using a special order five-laser BD Aria II high-speed cell sorter (BD Biosciences). Non-viable and hematopoietic cells were then excluded based on staining for DAPI and CD45, respectively. CD34^+^CD146^-^ adventitial cells and CD146^+^CD34^-^ pericytes were collected as PSC. Flow cytometry of purified samples was performed for the MSC markers CD44 and CD90 (BD Biosciences). Samples with detectable pericyte and adventitial populations were examined. Unless otherwise stated, cells were cultured in DMEM + 10% Fetal Bovine Serum (FBS) + 1% Penicillin/Streptomycin and medium was changed routinely every 3 days. N = 12 samples were examined. N = 9 human PSC samples were examined as a interspecies comparison, with tissues obtained under IRB approval.

### *In vitro* osteogenic differentiation assays

For osteogenic differentiation, cells were seeded onto 24-well plates at a density of 5x10^4^ cells/well. After attachment, cells were treated with osteogenic differentiation medium (ODM) consisting of DMEM, 10% FBS, 10 mM β-glycerophosphate and 50 μM ascorbic acid. In select studies, ODM was supplemented with dexamethasone (1 μM or 100 nM) or recombinant human NELL-1 (rhNELL-1, 0–1200 ng/ml) (Aragen Bioscience). The medium was refreshed every three days. After 14 days, osteogenic differentiation was assessed using alkaline phosphatase (ALP) staining, quantifications were normalized to total protein content[43]. Alizarin red (AR) staining and quantification was performed at 30 days according to manufacturer's protocol (Millipore, Billerica, MA).

Recombinant human NELL-1 was produced by Aragen Bioscience. Briefly, rhNELL-1 was produced by a genetically modified, DHFR-deficient CHO cell line. A series of CHO cell lines expressing rhNELL-1 were developed using classical DHFR gene amplification technology. After selection, in excess of 2,000 subclones were screened using a NELL-1 ELISA assay. From this screen, 12 clones were selected for expansion. Three clones that were observed to express the best were identified, and were used for all subsequent rhNELL-1 production.

### Quantitative real-time polymerase chain reaction (qRT-PCR)

Quantitative real-time polymerase chain reaction (qRT-PCR) reactions were run using the CFX96 Real-Time PCR detection system (Bio-Rad). Reactions were incubated in 96-well optical plates at 95°C for 10 minutes, followed by 40 cycles at 95°C for 15 s, and at 60°C for 60 s. The threshold cycle (Ct) data were determined using default threshold settings. The Ct was defined as the fractional cycle number at which the fluorescence passes the fixed threshold. Reactions were run in duplicate to triplicate per RNA isolate. Primer sequences are shown in S1 Table.

### Colony forming unit-fibroblast (CFU-F) assay

Cells were cultured and plated in six well plates at densities of 250, 500 and 1000 cells/well in standard growth medium. After 14 days, the number of colonies was measured and quantified after staining with Giemsa. Experiments were run in duplicates.

### Colony forming unit-osteoblast (CFU-OB) assay

Cells were seeded at a density of 250, 500 and 1000 cells/well in six-well plates and grown in standard growth medium. After attachment, medium was changed to osteogenic differentiation medium (ODM). ODM was constituted with 10 mM β-glycerophosphate and 50 μM ascorbic acid in DMEM + 10% FBS. After 14 days, the number of osteoblastic colonies was measured and quantified after ALP staining as previously described[10]. Experiments were run in duplicates.

### Trophic factor secretion

Canine PSC were seeded at 80% confluency in DMEM containing 10% FBS. After attachment, medium was changed to DMEM with 1% FBS and supplemented with NELL-1. Supernatant was harvested after 72 hours and Enzyme-linked immunosorbent assays (ELISA) for vascular endothelial growth factor (VEGF), platelet derived growth factor (PDGF), and fibroblastic growth factor-2 (FGF-2) were performed per the manufacturer’s instructions (BIOTANG, Waltham, MA). Experiments were run in duplicates.

### Statistical analysis

All results were expressed as mean ± standard error. Statistical analyses were performed using the SPSS16.0 software. All data were normally distributed. Student’s t test was used for two-group comparisons, and one-way ANOVA test was used for comparisons of 3 or more groups, followed by Tukey’s *post hoc* test. Differences were considered significant with *P < 0.05.

### Ethics statement

All research animals were handled according to the guidelines of the Chancellor’s Animal Research Committee for Protection of Research Subjects at the University of California, Los Angeles.

## Results

### Canine and human PSC are obtained by identical processes

**Fig 1:**
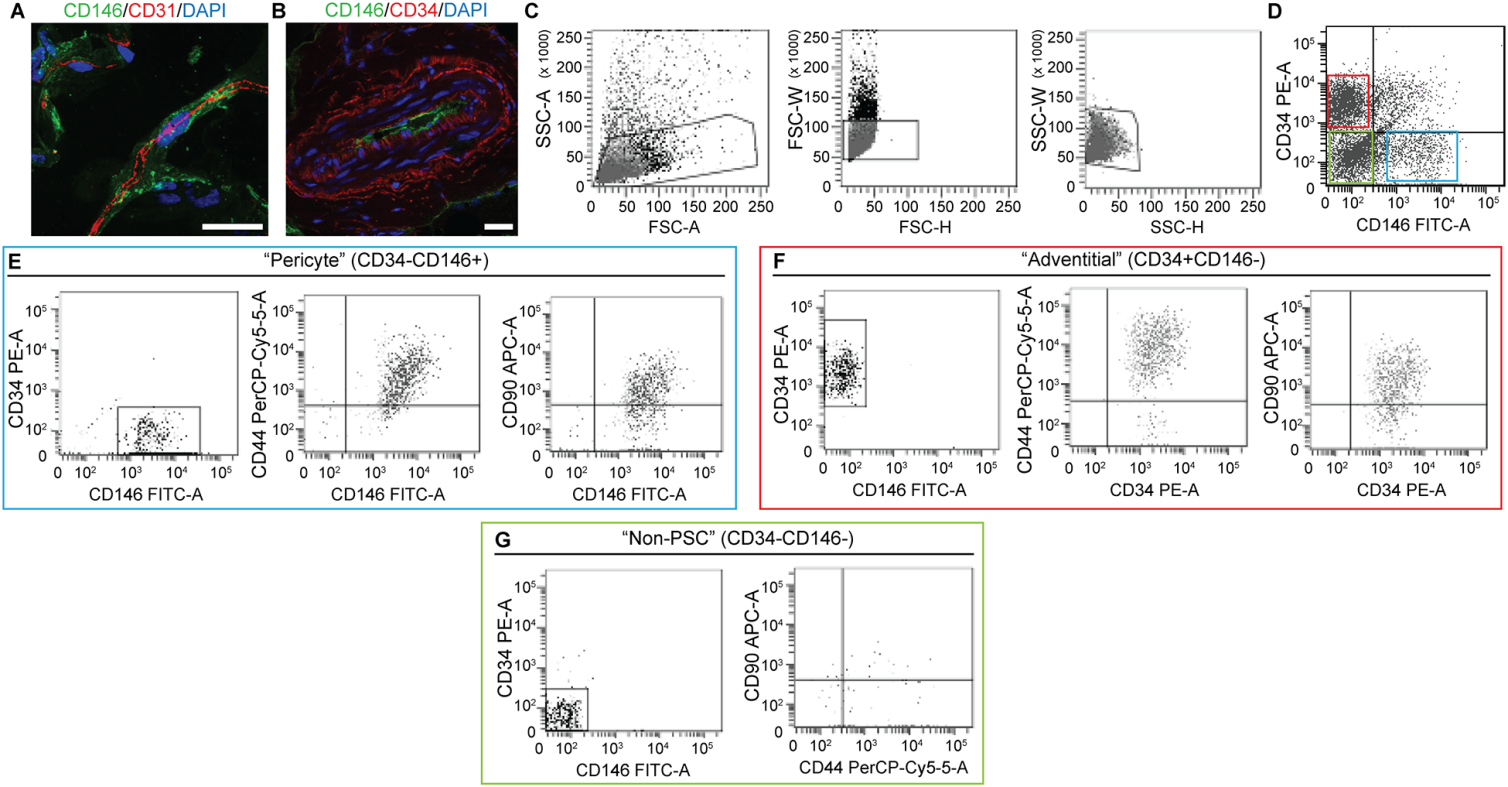
Canine PSC *in situ* identification. **Marker expression and isolation by fluorescence activated cell sorting (FACS). (A)** Canine adipose tissue stained with pericytes marker CD146 (green), endothelial marker CD31 (red) (63×), and **(B)** adventitial cell marker, CD34 (red), CD146 (green) (40×). DAPI nuclear counterstain appears blue. **(C)** Cell size. **(D)** Purified PSC consist of distinct CD34−CD146+ pericytes and CD34+CD146− adventitial cells. **(E)** CD146+CD34- pericytes and **(F)** CD146-CD34+ adventitial cells express characteristic MSC markers, including CD44 and CD90. In comparison, **(G)** CD146-CD34- “non-PSC” are predominantly negative for CD44 and CD90. Scale bar: 25 uM.

Previous studies have optimized the procedure for human PSC isolation[8, 9]. We sought to translate this protocol to canine tissues. First the cross reactivity and specificity of antibodies directed against human CD146 and CD34 were examined in canine adipose tissue (Fig 1). Results showed that previously used antibodies were cross reactive against canine PSC, including CD146 (Fig 1A) and CD34 (Fig 1B). Next, canine SVF was collected from enzymatically digested lipoaspirate. After the exclusion of DAPI+ dead cells and CD45+ hematopoietic cells, the detection of adventitial cells and pericytes was performed based on the differential expression of CD34 and CD146 (Fig 1D)[8, 9]. CD34+CD146− adventitial cells and CD146+CD34− pericytes were isolated by FACS in an identical manner to those derived from human adipose tissue, collectively referred to as PSC. We observed canine pericytes (Fig 1E) and adventitial cells (Fig 1F) to demonstrate positive expression of the MSC markers CD44 and CD90[44]. In comparison, the ‘non-PSC’ cell constituent showed rare CD44 and CD90 expression (Fig 1G).

### PSC are abundant in canine white adipose tissue

In order to obtain canine SVF, lipoaspirate was retrieved from twelve canine samples (Table 1). On average, cell viability of canine SVF was 65.7%, ranging from 30.2% to 93.6%. From this population, cells were further fractionated by FACS as adventitial cells and pericytes and collected together as PSC. On average, adventitial cells and pericytes comprised 20.1% and 9.7% of viable SVF, respectively. The composition of adventitial cells within SVF ranged from 6.4% to 39.8%, while pericytes were generally found in lower numbers, ranging from 2.1% to 18.8%. Total PSC therefore comprised on average 30.0% of viable SVF, ranging from 14.3% to 41.3%.

**Table 1:** Yield and frequency of PSC from canine adipose tissue.

Samples	SVF (million)	Viable SVF (%)	Adventitial (%)	Pericytes (%)	PSC (%)
1	4.5	62.0	14.7	6.9	21.6
2	1.0	63.2	17.7	11.2	28.9
3	24.0	93.6	21.4	9.9	31.3
4	16.6	64.0	29.5	8.0	37.5
5	6.2	76.9	8.5	18.1	26.6
6	8.4	84.0	24.1	14.2	38.3
7	20.0	30.2	6.4	7.9	14.3
8	12.6	62.7	11.6	12.5	24.1
9	50.0	61.5	39.8	1.5	41.3
10	24.8	65.7	9.3	18.8	28.1
11	57.6	55.3	24.2	5.7	29.9
12	60.0	69.2	33.8	2.1	35.9
**Average ± SEM**	23.8 ± 6.0	65.7 ± 4.5	20.1 ± 3.1	9.7 ± 1.6	30.0 ± 2.2

**Fig 2:**
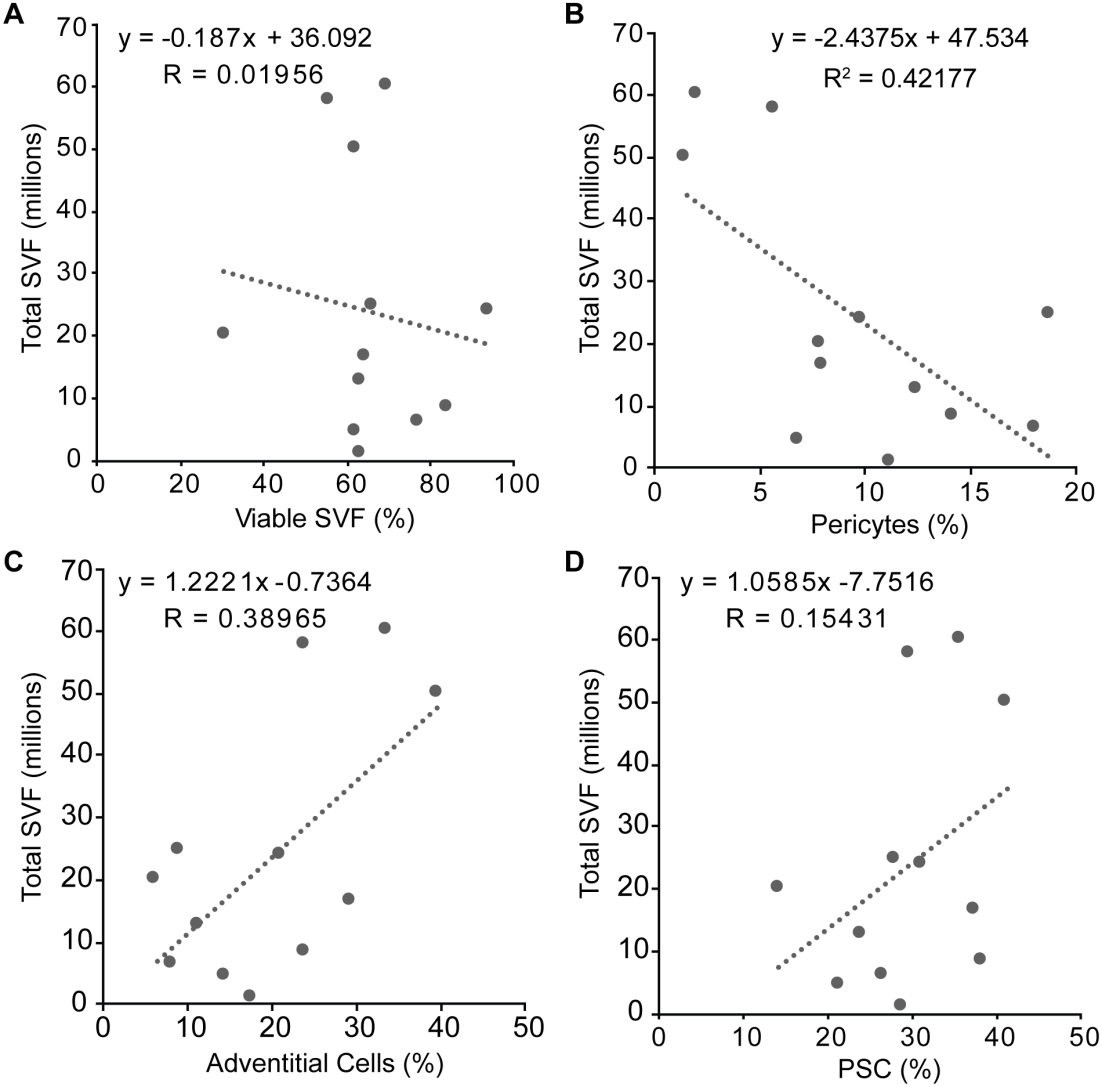
Comparison of total SVF to PSC. Each sample is represented by a single data point. Linear fit of total SVF cell yield with respect to **(A)** percent viable SVF, **(B)** percent pericytes, **(C)** percent adventitial cells, and **(D)** percent PSC. Trendlines, equations, and R^2^ values are shown. Little or no linear correlation was observed across all variables examined.

We next sought to determine how SVF yield or viability affected parameters of PSC harvest. Scatter plots with linear fit trendlines revealed little or no correlation across all parameters examined (Fig 2). Specifically, a vague and weak negative correlation (R^2^ = 0.38965) between the total SVF isolate and the percentage of viable SVF was observed (Fig 2A). Total yield of SVF isolate was then compared with PSC yield by cell type (Fig 2B–2D). The percentage of pericytes showed a weak negative correlation with total SVF numbers (R^2^ = 0.42177) (Fig 2B). In contrast, the percentage of adventitial cells demonstrated a vague and weak positive correlation with total SVF numbers (R^2^ = 0.38965) (Fig 2C). Thus, the total PSC population had no clear linear relationship with total SVF numbers (R^2^ = 0.15431) (Fig 2D).

### Canine PSC demonstrate MSC characteristics

**Fig 3:**
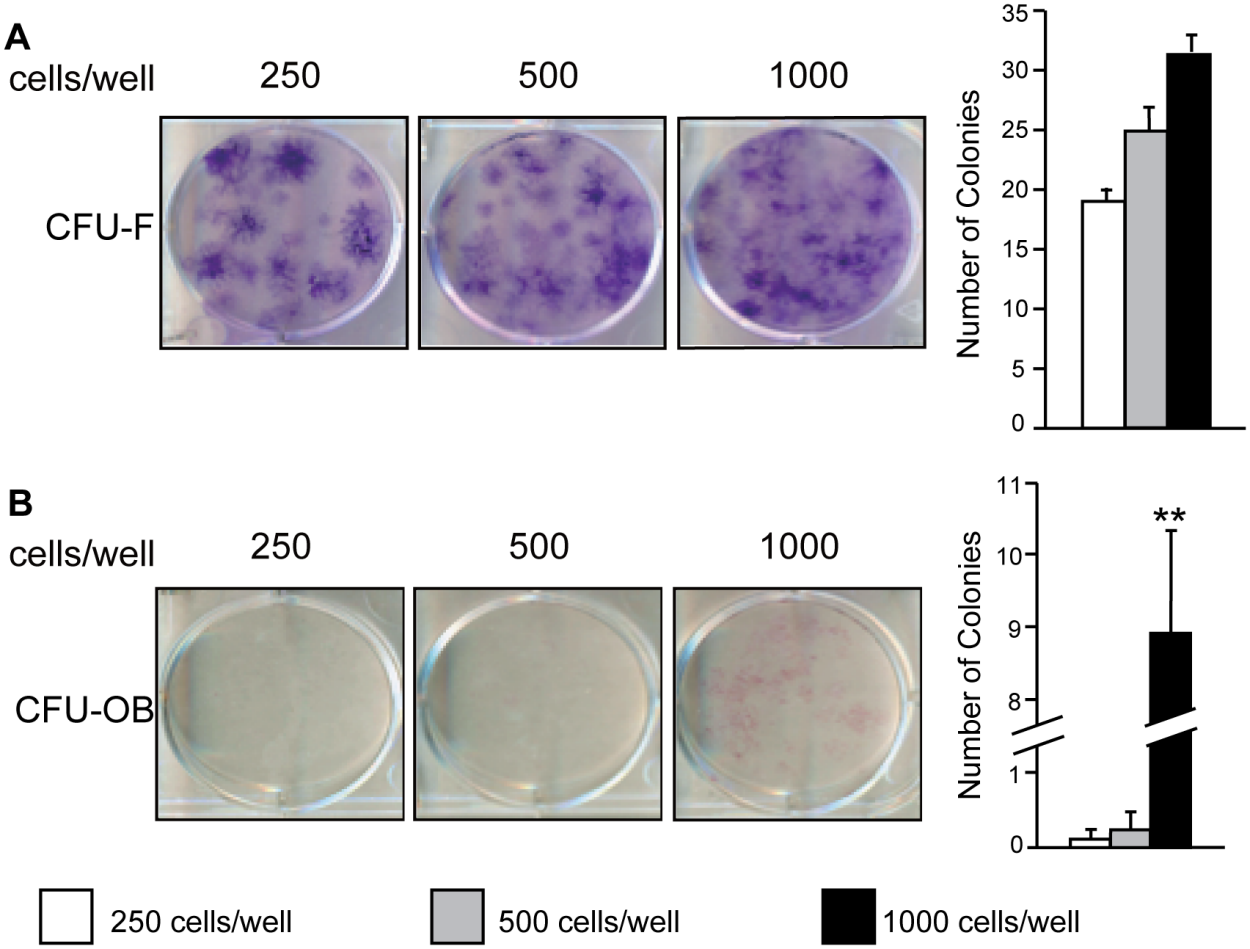
Canine PSC retain stem cell character following fluorescence activated cell sorting (FACS). Freshly isolated PSC were seeded at low densities (200, 500, and 1,000 cells/well) in 6 well plates and evaluated for colony formation after 14 days. **(A)** Clonal colonies formed by colony forming units-fibroblast (CFU-F) were observed by giemsa staining and **(B)** clonal colonies formed by colony forming units-osteoblast (CFU-OB), visualized by ALP staining after 14 days of osteogenic differentiation are shown. Mean ± SEM of total colony forming units are shown. *p < 0.05, **p < 0.01 versus 250 cells/ well.

Canine PSC were next evaluated based on their ability to form clonal fibroblastic colonies at low seeding densities. As expected, PSC formed more total fibroblastic colonies as seeding densities increased. 250 cells, 500 cells, and 1,000 cells were able to form an average of 19, 25, and 32 colonies, respectively (Fig 3A). At higher density, colonies began to merge with one another, preventing an accurate colony count. In order to measure the ability of PSC to form osteoblastic colonies *in vitro*, cells were cultured with supplemental osteogenic factors ascorbic acid and β-glycerophosphate. Canine PSC readily formed osteoblastic colonies when seeded at a density of 1,000 cells/well (Fig 3B). This was observed by the formation of 0.13, 0.25, and 9 colonies on average by 250 cells, 500 cells, and 1,000 cells, respectively. Thus, like human PSC, canine PSC have MSC characteristics including the ability to form fibroblastic and osteoblastic colonies.

### NELL-1 enhances canine PSC osteogenic differentiation

The effects of different contents of osteogenic differentiation medium (ODM) on canine PSC were first assessed, including the addition of two different concentrations of dexamethasone (S1 Fig). As expected, all ODM types (with or without dexamethasone) increased the osteogenic differentiation of PSC in comparison to growth medium control. Overall, dexamethasone addition to ODM did not result in a reliable increase in markers of osteogenic differentiation, although higher concentration dexamethasone significantly increased gene expression of *COL1A1*.

**Fig 4:**
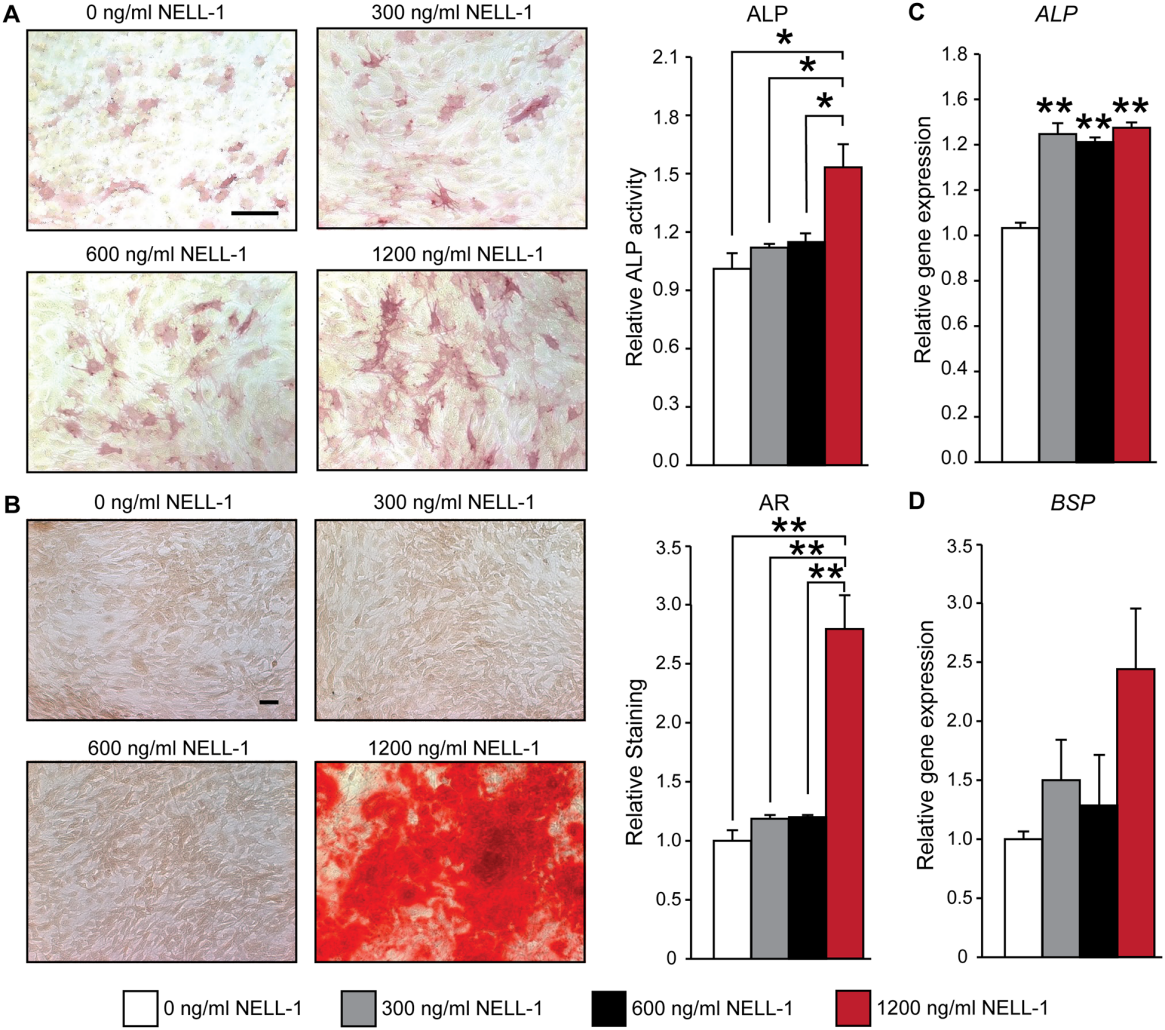
Canine PSC are responsive to the osteoinductive factor NELL-1. Canine PSC were cultured under osteogenic conditions and treated with varying doses of NELL-1 (0, 300, 600, 1200 ng/mL). Osteogenic differentiation was evaluated by **(A)** alkaline phosphatase (ALP) staining and enzyme activity at 5 days and **(B)** Alizarin Red (AR) staining at 14 days of osteogenic differentiation. Quantification of the AR stain is based on random *n* = 3 (40×) images, using the Adobe Photoshop cc 2016 magic wand tool (tolerance = 30). Scale Bar = 200 μM. Gene expression analysis of osteogenic differentiation markers **(C)** *ALP* and **(D)** *BSP* was performed after 14 days of osteogenic differentiation. Mean ± SEM are shown. *p < 0.05, **p < 0.01.

Having observed that Dexamethasone to not reliably influence canine PSC osteogenesis, we next examined the effects of recombinant NELL-1 protein. In order to examine the osteogenic effects of the differentiation factor NELL-1 on canine PSC, cells were treated with recombinant protein in increasing dosages of 300, 600, and 1,200 ng/mL and osteogenic effects were visualized by alkaline phosphatase (ALP) and Alizarin red (AR) staining (Fig 4). ALP activity was slightly increased by the addition of NELL-1 at concentrations of 300 and 600 ng/mL by 12.1% and 15.3%, respectively. In contrast, 1,200 ng/mL supplementation of NELL-1 to canine PSC stimulated a stronger osteogenic response observed by a 55.7% increase in ALP activity (Fig 4A). Similarly, bone nodule deposition quantified by AR staining only increased by 18.6% and 20.1% after the supplementation of 300 ng/mL and 600 ng/mL NELL-1, respectively, while the addition of 1,200 ng/mL increased bone nodule deposition 284% over the control (Fig 4B). Osteogenic gene expression among PSC treated with or without NELL-1 was next assayed by qRT-PCR (Fig 4C and 4D). Expression of the osteogenic markers *Alkaline Phosphatase* (*ALP)* and *Bone Sialoprotein* (*BSP)* showed an increase in response to the addition of NELL-1 at all doses (Fig 4C and 4D), although these effects were not statistically significant with *BSP*. These data demonstrate that canine PSC are responsive to the osteoinductive protein NELL-1 in a dose-dependent fashion.

### NELL-1 enhances canine PSC production of trophic factors VEGF, FGF-2, and PDGF

**Fig 5:**
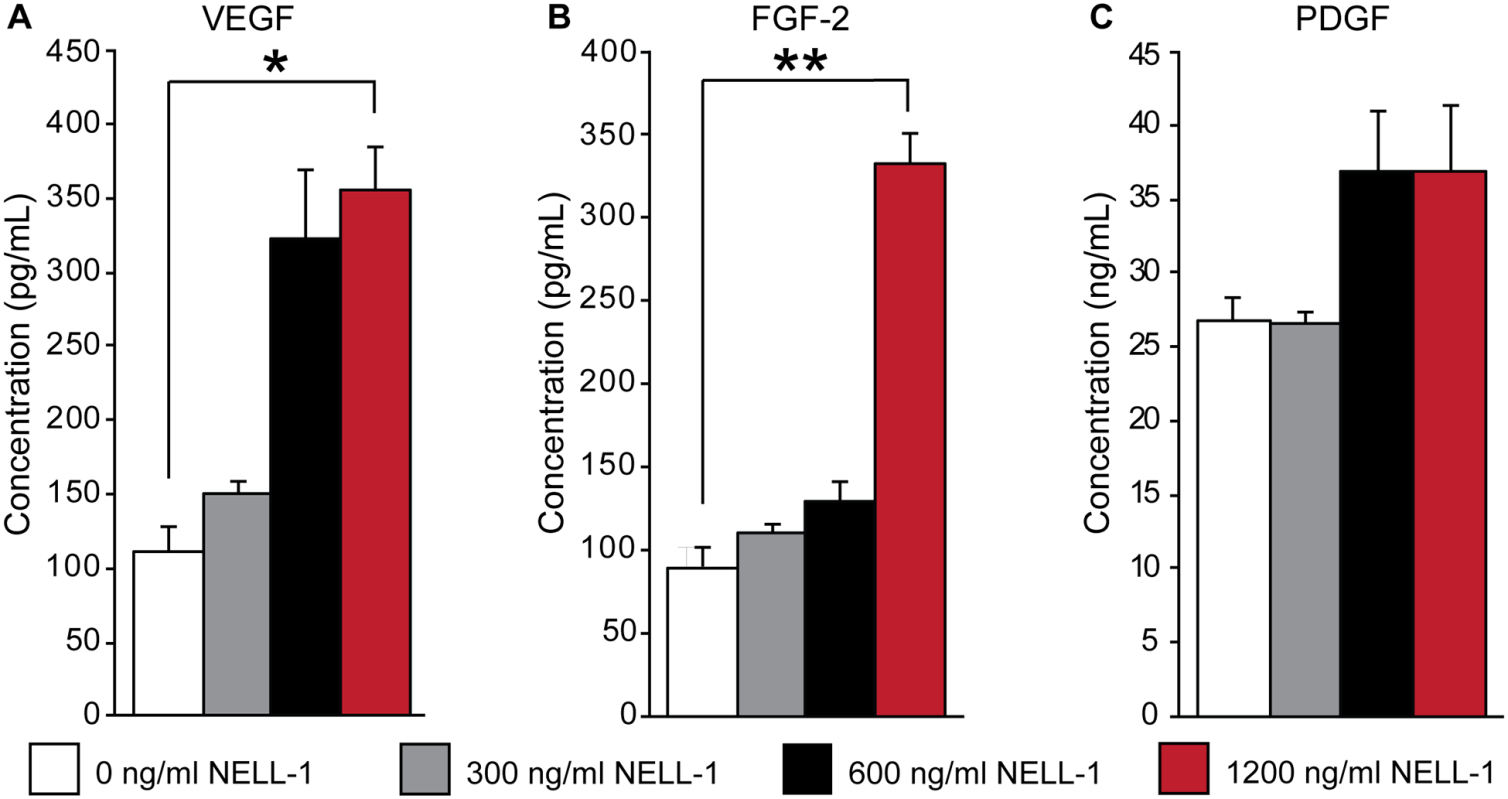
Secretion of trophic factors by canine PSC in response to NELL-1. Secreted **(A)** vascular endothelial growth factor (VEGF), **(B)** fibroblastic growth factor-2 (FGF-2), and **(C)** platelet derived growth factor (PDGF) in the conditioned medium as assessed by enzyme-linked immunosorbent assay (ELISA) at 72-h treatment with NELL-1 (300–1,200 ng/mL). Mean ± SEM are shown. *p < 0.05, **p < 0.01.

PSC, like other MSC types, are hypothesized to exert their effects via both direct osteogenic differentiation and paracrine stimulation of osteogenesis and vasculogenesis[45]. In order to evaluate the effects of NELL-1 on trophic factor production in canine PSC, enzyme-linked immunosorbent assays for the angiogenic factors: vascular endothelial growth factor (VEGF), fibroblast growth factor-2 (FGF-2), and platelet-derived growth factor (PDGF) were used to detect protein production in response to NELL-1 (300, 600, and 1,200 ng/mL). The production of VEGF was enhanced by addition of NELL-1 in a dose dependent manner in canine PSC, as the addition of NELL-1 increased the production of VEGF by 33.8% to 320.9% (Fig 5A). Similarly, FGF-2 production was enhanced by the addition of NELL-1. The addition of 1,200 ng/mL NELL-1 increased FGF-2 production by 369.4% over the control (Fig 5B). PDGF production in canine PSC was modestly increased by the addition of NELL-1 up to 37% (Fig 5C). In summary, NELL-1 induces canine PSC expression of modulate production of osteogenic and angiogenic growth and differentiation factors.

## Discussion

The current study investigates canine PSC as a stem/stromal cell population for translational efforts in bone tissue engineering. Our data revealed that canine PSC are readily isolated from SVF in abundant numbers with an identical isolation protocol as human PSC. These data further confirm the perivascular location of MSC in mammals, as already described in man[13, 46, 47], mouse[48, 49], sheep[50] and horse (Esteves *et al*., *under review*). Remarkably, the same markers were used to identify PSC in these different species, using in most instances cross-reactive antibodies. This suggests a highly conserved set of perivascular markers for an ancestral population of tissue regenerative cells. Additionally, we found that canine PSC secrete the trophic factors VEGF, FGF-2, and PDGF and are responsive to the novel osteoinductive molecule NELL-1.

Important similarities and differences exist between canine and human PSC. Similarities between canine and human sources include their MSC properties, ability to undergo osteogenic differentiation, and responsiveness to osteoinductive cues like NELL-1[10]. Like human PSC, canine PSC express MSC markers such as CD44 and CD90, and give rise to clonal fibroblastic cell colonies at low densities. Also similar to human PSC, canine PSC were able to form osteoblastic colonies and undergo the process of osteogenic differentiation / mineralization *in vitro*. Finally, both human and canine cells appear to respond to the pro-osteogenic stimulus of NELL-1 protein[15].

Despite the similarities between PSC of canine or human origin, differences also exist. These differences include: relative cell abundance within adipose tissue, and baseline osteogenic potential. In our study of twelve samples, canine PSC represent ~30% of canine SVF. This contrasts with human PSC which represent ~39–41% of human SVF[9, 10]. When these differences are further examined, it appears that the difference can be attributable to the frequency of adventitial cell subpopulation among canine samples. Pericyte frequency is similar between species, representing 9.7% and 8% from canine and human SVF, respectively[9]. Adventitial cell frequency appears dissimilar between species, representing 20.1% and 33% from canine and human SVF[9]. Whether this reflects an underlying difference in the vascularity of canine adipose tissue, a difference in the relative adventitial cell density among larger vessels, or a difference in antibody affinity based on species is not yet known. A second difference is relative frequency of MSC markers in dog versus human cells. A relative consensus exists regarding the minimal criteria for multipotent mesenchymal stem/stromal cells (MSC) in human cells, as put forth by Dominici et al.[51]. Unfortunately, a similar consensus has not been reached for canine MSC. However, our results lie in similarity to most prior reports from other research groups in canine adipose-derived MSC, which by and large report a CD44+CD90+CD45- cell population as the most consistent markers for canine MSC[52–58]. Another and more striking difference between PSC by species is their baseline osteogenic potential (S2 Fig). We previously reported that human PSC undergo brisk osteogenic differentiation over the course of two weeks or less in standard osteogenic culture with only ascorbic acid and β-glycerophosphate supplementation[10]. In contrast, the present study showed that canine PSC after similar isolation and propagation techniques undergo a much slower process of osteogenic differentiation, with very sparse bone nodules formed. Only with NELL-1 were significant bone nodules seen. These results are in agreement with prior comparative findings across species using unpurified adipose-derived stromal cells (ASC)[59]. In this study, canine ASC were found to proliferate to a greater degree than human ASC, but had much reduced osteogenic potential. Canine ASC were also responsive to the pro-osteogenic effects of retinoic acid supplementation, while human ASC were not[60]. These studies using an unpurified stromal population clearly have overlapping findings with purified, adipose-tissue derived PSC. As canine PSC are translated into an *in vivo* model of bone repair, their relatively low baseline osteogenic potential must be taken into account.

Although efforts in canine PSC isolation and application represent a step forward to clinical translation of a PSC based product for skeletal regeneration in patients, use of canine tissue poses several practical concerns that should be addressed. First and foremost, the societal view of dogs as a pet and human companion prompts use of alternate large animal models. Alternative large animals that do not have the same societal significance, such as sheep, offer many similarities in bone structure and composition to human bone[61–64]. Of note, a similar PSC population has recently been successfully isolated from ovine adipose tissue[50]. Importantly, PSC isolation has potential translational applicability in both human and veterinary medicine[65]. The therapeutic applications of PSC may well be beneficial for pets as well as man.

In summary, our data show that canine PSC are abundant in the SVF in clinically relevant numbers, comprising on average 30% of the viable SVF. Importantly, canine PSC retain MSC characteristics, and are responsive to osteoinductive stimuli such as NELL-1. Although differences in baseline osteogenic potential exist between canine and human PSC, use of FACS purified canine PSC represents a new method to deliver autologous cells in a large animal model of bone repair to evaluate strategies/tactics to be developed in humans.

## Supporting information

S1 Data Raw data file. (XLSX)

S1 Fig Effects of dexamethasone on osteogenic differentiation of canine PSC. Canine PSC were cultured under osteogenic conditions and treated with varying doses of dexamethasone (100 nM or 1 μM). Osteogenic differentiation was evaluated by **(A)** alkaline phosphatase (ALP) staining and **(B)** quantification at 7 days. Quantification of the ALP stain is based on random *n* = 10 (40×) images, using the Adobe Photoshop cc 2016 magic wand tool (tolerance = 30). Scale bar = 100 μM. Gene expression analysis of osteogenic differentiation markers **(C)** *ALP* **(D)** *COL1A1* and **(D)** *OPN* was performed on day 14 by qRT-PCR. Mean ± SEM are shown. *p < 0.05, **p < 0.01.
(TIF)

S2 Fig Differences in human and canine PSC baseline osteogenic potential. Human and canine PSC were cultured under identical osteogenic conditions and stained by **(A)** alkaline phosphatase (ALP) and **(B)** alizarin red (AR). Quantification of the AR and ALP stains are based on random *n* = 4–9 (40×) images, using the Adobe Photoshop cc 2016 magic wand tool (tolerance = 30). Mean ± SEM are shown. *p < 0.05, **p < 0.01.
(TIF)

S1 Table qRT-PCR primers. (DOCX)
