# Supplementary figures and images for: Isolation and characterization of canine perivascular stem/stromal cells for bone tissue engineering
Source: PLoS One. 2017 May 10;12(5):e0177308. doi: 10.1371/journal.pone.0177308 (PMC5425216; doi:10.1371/journal.pone.0177308)

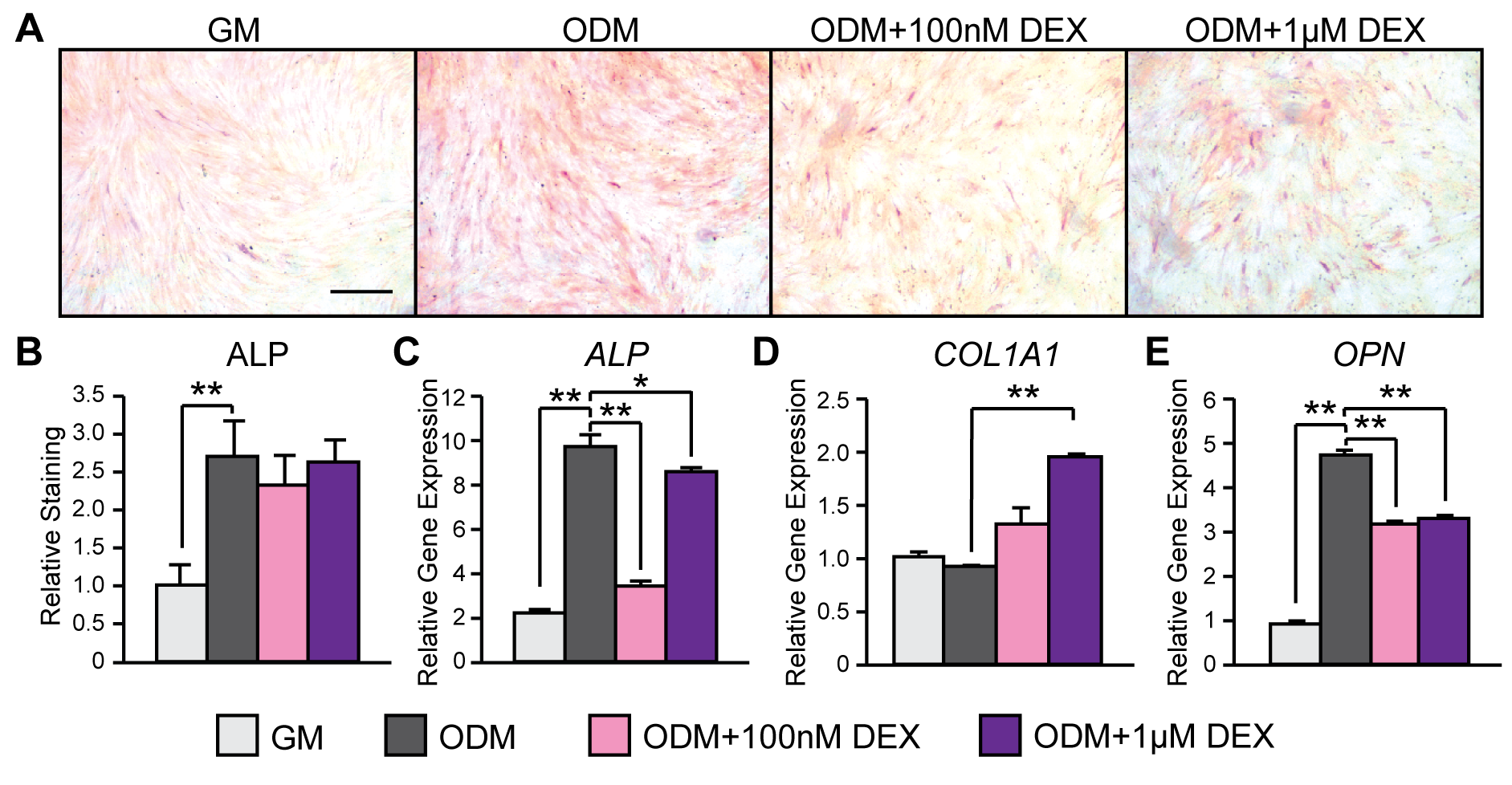

Supplement: S1 Fig — Canine PSC were cultured under osteogenic conditions and treated with varying doses of dexamethasone (100 nM or 1 μM). Osteogenic differentiation was evaluated by (A) alkaline phosphatase (ALP) staining and (B) quantification at 7 days. Quantification of the ALP stain is based on random n = 10 (40×) images, using the Adobe Photoshop cc 2016 magic wand tool (tolerance = 30). Scale bar = 100 μM. Gene expression analysis of osteogenic differentiation markers (C) ALP (D) COL1A1 and (D) OPN was performed on day 14 by qRT-PCR. Mean ± SEM are shown. *p < 0.05, **p < 0.01. (TIF) [file pone.0177308.s002.tif]

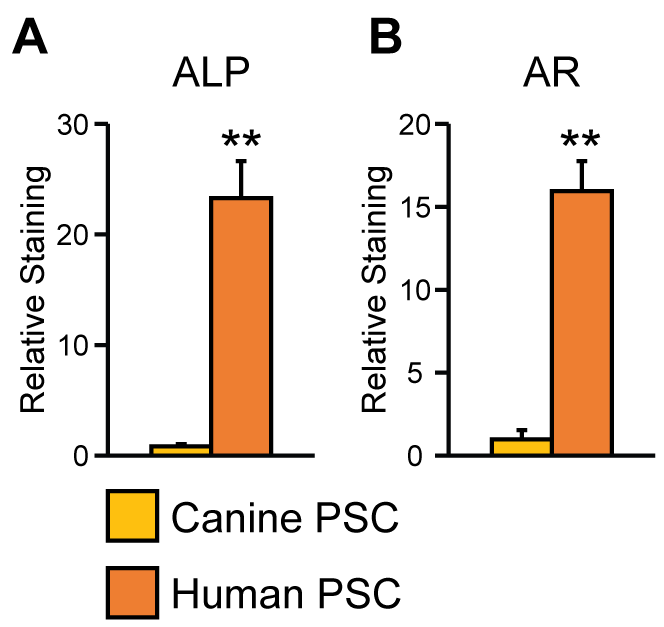

Supplement: S2 Fig — Human and canine PSC were cultured under identical osteogenic conditions and stained by (A) alkaline phosphatase (ALP) and (B) alizarin red (AR). Quantification of the AR and ALP stains are based on random n = 4–9 (40×) images, using the Adobe Photoshop cc 2016 magic wand tool (tolerance = 30). Mean ± SEM are shown. *p < 0.05, **p < 0.01. (TIF) [file pone.0177308.s003.tif]
