# Supplementary material for: Isolation and characterization of canine perivascular stem/stromal cells for bone tissue engineering
Source: PLoS One. 2017 May 10;12(5):e0177308. doi: 10.1371/journal.pone.0177308 (PMC5425216; doi:10.1371/journal.pone.0177308)
Supplement: S1 Table — (DOCX) [file pone.0177308.s004.docx]

**Table S1 - qRT-PCR primers**

| Gene | Forward | Reverse |
| --- | --- | --- |
| *ALP* | 5’-TTC AAA CCG AGA CAC AAG CAC T-3’ | 5’-GGG TCA GTC ACG TTG TTC CTG T-3’ |
| *BSP* | 5’-TTG CTC AGC ATT TTG GGA ATG G-3’ | 5’-AAC GTG GCC GAT ACT TAA AGA CC-3’ |
| *COL1A1* | 5’-CCA AGA AGA AGA CAT CCC ACC-3’ | 5’-GAT CAC GTC ATC GCA CAA CA-3’ |
| *GAPDH* | 5’-CGG GCG TTG ATG ACA AGT TTC CCG-3’ | 5’-CTA CCC ACG GCA AAT TCC AC-3’ |
| *OPN* | 5’-CGA GTC TGA TGA ATC CGA TGA A-3’ | 5’-TTG GGT TGC TGG AAT GTC AGT-3’ |
